# The reliability of the physical examination to guide fluid therapy in adults with severe falciparum malaria: an observational study

**DOI:** 10.1186/1475-2875-12-348

**Published:** 2013-10-01

**Authors:** Josh Hanson, Sophia WK Lam, Shamsul Alam, Rajyabardhan Pattnaik, Kishore C Mahanta, Mahatab Uddin Hasan, Sanjib Mohanty, Saroj Mishra, Sophie Cohen, Nicholas Day, Nicholas White, Arjen Dondorp

**Affiliations:** 1 Mahidol Oxford Tropical Medicine Research Unit, Faculty of Tropical Medicine, Mahidol University, Bangkok, Thailand; 2 Menzies School of Health Research, Darwin, Northern Territory, Australia; 3 Chittagong Medical College Hospital, Chittagong, Bangladesh; 4 Ispat General Hospital, Rourkela, India; 5 Academic Medical Center, University of Amsterdam, Amsterdam, The Netherlands; 6 Nuffield Department of Medicine, Centre for Tropical Medicine, University of Oxford, Oxford, UK

**Keywords:** Severe malaria, Physical examination, Fluid resuscitation

## Abstract

**Background:**

Adults with severe malaria frequently require intravenous fluid therapy to restore their circulating volume. However, fluid must be delivered judiciously as both under- and over-hydration increase the risk of complications and, potentially, death. As most patients will be cared for in a resource-poor setting, management guidelines necessarily recommend that physical examination should guide fluid resuscitation. However, the reliability of this strategy is uncertain.

**Methods:**

To determine the ability of physical examination to identify hypovolaemia, volume responsiveness, and pulmonary oedema, clinical signs and invasive measures of volume status were collected independently during an observational study of 28 adults with severe malaria.

**Results:**

The physical examination defined volume status poorly. Jugular venous pressure (JVP) did not correlate with intravascular volume as determined by global end diastolic volume index (GEDVI; r_s_ = 0.07, p = 0.19), neither did dry mucous membranes (p = 0.85), or dry axillae (p = 0.09). GEDVI was actually higher in patients with decreased tissue turgor (p < 0.001). Poor capillary return correlated with GEDVI, but was present infrequently (7% of observations) and, therefore, insensitive. Mean arterial pressure (MAP) correlated with GEDVI (r_s_ = 0.16, p = 0.002), but even before resuscitation patients with a low GEDVI had a preserved MAP. Anuria on admission was unrelated to GEDVI and although liberal fluid resuscitation led to a median hourly urine output of 100 ml in 19 patients who were not anuric on admission, four (21%) developed clinical pulmonary oedema subsequently. MAP was unrelated to volume responsiveness (p = 0.71), while a low JVP, dry mucous membranes, dry axillae, increased tissue turgor, prolonged capillary refill, and tachycardia all had a positive predictive value for volume responsiveness of ≤50%. Extravascular lung water ≥11 ml/kg indicating pulmonary oedema was present on 99 of the 353 times that it was assessed during the study, but was identified on less than half these occasions by tachypnoea, chest auscultation, or an elevated JVP. A clear chest on auscultation and a respiratory rate <30 breaths/minute could exclude pulmonary oedema on 82% and 72% of occasions respectively.

**Conclusions:**

Findings on physical examination correlate poorly with true volume status in adults with severe malaria and must be used with caution to guide fluid therapy.

**Trial registration:**

Clinicaltrials.gov identifier: NCT00692627

## Background

After several days of fever, sweating, vomiting and, frequently, impaired consciousness, adults with severe malaria are usually dehydrated and hypovolaemic - some profoundly so [1]. As hypovolaemia evolves, the Frank-Starling principle dictates that the cardiac output will fall, reducing tissue perfusion and potentially exacerbating the metabolic acidosis and acute kidney injury (AKI) that are strong predictors of mortality [2,3]. Although microvascular sequestration and endothelial dysfunction are central to the pathogenesis of acidosis and AKI in severe malaria [4-6], neither have an effective treatment [7]. Meanwhile hypovolaemia has a widely available and inexpensive remedy: fluid resuscitation.

If a patient is volume responsive, fluid loading increases stroke volume and hence cardiac output and oxygen delivery to tissues. However in severe malaria, even if rehydration improves systemic haemodynamics, there may be limited improvement in the microvascular obstruction, which makes a greater relative contribution to tissue hypoperfusion [1,8]. Adults with severe malaria also have a generalized increase in vascular permeability which is particularly important in the lungs where pulmonary oedema can occur rapidly, unpredictably, and is frequently fatal [8-10]. The hazards of fluid loading have also been demonstrated recently in African children with severe malaria, with mortality substantially higher in the patients receiving liberal resuscitation than in those receiving standard maintenance therapy [11]. Finding a balance between over- and under-hydration is therefore challenging for the clinician managing severe malaria.

As patients with severe malaria will usually be managed in a resource-poor setting, World Health Organization (WHO) guidelines necessarily emphasize clinical assessment of volume status to guide rehydration [12]. These guidelines emphasize the utility of blood pressure, urine output and jugular venous pressure (JVP) in particular [12], although there are few study data to support the safety of this approach.

In order to assess the reliability of physical examination in guiding the fluid management of adults with severe malaria, data from a recent prospective study assessing fluid resuscitation of adults with severe falciparum malaria were examined. The objective was to determine the relationship between the findings on the patient’s physical examination and their volume status. Did simple clinical signs identify hypovolaemic patients and, of critical clinical relevance, did these signs identify patients who were volume responsive or at risk of pulmonary oedema?

## Methods

Patients were recruited at Chittagong Medical College Hospital in Bangladesh and Ispat General Hospital in Rourkela, India in 2008. Patients were defined as having malaria if asexual forms of *Plasmodium falciparum* were present on blood film or, if expert microscopy was not immediately available, an immunochromatographic rapid diagnostic test (Paracheck Pf, Orchid Biomedical Systems, India) was positive. Falciparum malaria was later confirmed by examination of a simultaneously collected blood slide. As invasive haemodynamic monitoring was employed, only severely ill adult patients with malaria were enrolled. The prospectively defined severity criteria were: peripheral venous base deficit of >6 mmol/L, blood urea nitrogen >60 mg/dL (21.4 mmol/L) or clinical pulmonary oedema (defined as oxygen saturation <90% with bi-basal crepitations on respiratory examination). Patients were enrolled only after written informed consent was obtained from an accompanying relative via a local translator. Patients were excluded if they were less than 16 years of age or if they had received adequate anti-malarial treatment for more than 24 hours before enrolment.

### Ethical review

The Bangladeshi Medical Research Council, the institutional ethical board of Ispat General Hospital, and the Oxford Tropical Research Ethics Committee provided ethical approval for the study. Regular safety reviews were performed by independent local committees and all deaths and serious adverse events were reported to these committees within 24 hours for appraisal.

### Patient management

All patients were admitted to an intensive care unit where a detailed history was taken and a physical examination was performed. Patients received intravenous artesunate and supportive care as per treatment guidelines of the time [13]. The original study assessed the response to liberal fluid resuscitation guided by published algorithms using volumetric indices [14]. Patients received normal (0.9%) saline intravenous fluid replacement and were assessed hourly during the first six hours and then six hourly thereafter until 96 hours, the patient’s death or a patient/family request to discontinue monitoring, whichever came first. Blood was transfused if the haemoglobin concentration fell below 5 g/dL. Respiratory support, dialysis, and inotropic support were initiated based on the judgement of the attending clinicians.

### Investigations

#### Haemodynamic investigations

Haemodynamic measurements were made using transpulmonary thermodilution and arterial pulse contour analysis (PiCCO-plus®, Pulsion, Germany). To obtain these measurements a standard triple lumen central venous catheter was inserted into the subclavian or internal jugular vein and a 5-French thermistor tipped arterial catheter (Pulsiocath, Pulsion, Germany) was inserted into the femoral artery. The physiological basis for measurement of haemodynamic variables using the PiCCO system is described in detail elsewhere [15,16]. Briefly, chilled saline (<8 C) was injected into the central vein, and the thermistor at the tip of the femoral artery catheter measured the downstream temperature change. Cardiac output was calculated by analysis of the thermodilution curve using a modified Stewart-Hamilton algorithm. All volumetric parameters were obtained by analysis of the thermodilution curve. These parameters include global end diastolic volume (GEDVI), a measure of volume status, and extravascular lung water (ELWI). The patient’s height was entered into the PiCCO monitor that calculated the ideal body weight and indexed values. The target GEDVI was >700 ml/m^2^ while aiming to maintain the ELWI ≤10 ml/kg. Central venous pressure (CVP) was measured using a manometer connected to the patient and a flask of saline via a three-way tap, the level of the right atrium was designated as the zero point. Patients were defined as volume responsive if there was a greater than 15% increase in cardiac index with a fluid load of greater than 500 ml. In this study, the ELWI cut-off for pulmonary oedema was set at ≥11 ml/kg based on recent autopsy validation studies [17].

#### Clinical assessment

Before each invasive haemodynamic evaluation, one of two clinicians (JH and SWKL, both with over five years’ post-graduate clinical experience) performed a physical examination with a particular emphasis on the patient’s volume status. The mucous membranes were classified as normal, mildly dry, or very dry and the axillae that were classified as moist or dry. Capillary refill was assessed by applying finger pulp pressure and classified as normal (<2 seconds), prolonged (2–4 seconds) or very prolonged (>4 seconds). Tissue turgor was determined by examining the skin on the abdomen and was classified as normal or decreased. JVP was assessed with the patient at 45º and was recorded as the height above the sternal angle. It was classified as very low (absent on inspection, but filling of neck veins confirmed with compression at the base of the neck), low (1–2 cm above the sternal angle), normal (3–4 cm above the sternal angle), or elevated (≥5 cm above the sternal angle). Blood pressure and heart rate were recorded from the intra-arterial catheter. Respiratory rate was counted at the bedside for 30 seconds. The lungs were auscultated and crepitations were classified as absent, mild (lower third) moderate (lower 2/3) or severe (entire lung field).

#### Statistical analysis

Data were collected and entered into an anonymized database and analysed using statistical software (Stata version 10, StataCorp). Correlation coefficients were determined using Spearman’s method. Groups were analysed using the Kruskal-Wallis test. Multivariate linear regression was used to determine the relative contributions of explanatory variables when more than one was significant in univariate analysis.

## Results

Twenty eight patients were enrolled and 23 survived to discharge. All patients were hypovolaemic on enrolment: GEDVI (median 481 ml/m^2^, range 346–675 ml/m^2^, normal 680–800 ml/m^2^). Two patients had clinical pulmonary oedema on enrolment and so did not receive liberal fluid resuscitation. Of the remaining 26 patients, 23 (88%) had a severe metabolic acidosis while nine (35%) satisfied the AKI criterion. The 26 patients that were resuscitated received 81 fluid boluses of greater than 500 ml (a median of three per patient) without concurrent inotropes during their hospitalization. The cardiac output was volume responsive on 23 of these 81 occasions. Eight of the 26 patients developed clinical pulmonary oedema during their hospital stay.

### Assessment of hypovolaemia and fluid responsiveness

#### Jugular venous pressure

The JVP correlated with the invasively measured CVP, on admission (r_s_ = 0.45, p = 0.02) and the 367 times both were assessed simultaneously (r_s_ = 0.43, p < 0.001) over the course of the study. However the JVP did not correlate significantly with the intravascular volume (as determined by the GEDVI) on either admission (r_s_ = 0.32, p = 0.11) nor on the 367 times they were measured concurrently (r_s_ = 0.07, p = 0.19). Mean GEDVI was similar in the patients with a very low JVP and those with an elevated JVP (Table 1). There was no relationship between JVP and cardiac index (CI) on admission (r_s_ = 0.04, p = 0.85), nor on the 363 times they were concurrently measured in the absence of inotropic support (r_s_ = 0.03, p = 0.85). The JVP performed poorly in identifying on which of these occasions patients would be fluid responsive: a low JVP had a positive predictive value (PPV) of 42% (95% confidence intervals (CI): 23-63%), while a very low JVP had a PPV of 40% (95% CI: 14-68%).

**Table 1:** Correlation between jugular venous pressure and invasive haemodynamic measures

	**Number of observations**	**GEDVI**	**CI**^**2**^	**ELWI**^**3**^
		**mean (95% CI)**^**1**^	**mean (95% CI)**	**mean (95% CI)**
Very low JVP ^4^	68	566 (535 – 598)	3.39 (3.24 – 3.54)	10.5 (9.4 – 11.6)
Low JVP ^5^	50	519 (497–540)	3.38 (3.21 – 3.55)	9.6 (8.9 – 10.2)
Normal JVP ^6^	74	599 (570 – 629)	3.58 (3.42 – 3.75)	9.0 (8.5 – 9.5)
Elevated JVP ^7^	175	570 (553–588)	3.46 (3.36 – 3.57)	9.4 (9.0 – 9.8)

^1^ Global end diastolic volume index (normal range 680–800 ml/m^2^).

^2^ Cardiac index (normal range 2.5-4.0 L/min/m^2^).

^3^ Extravascular Lung Water Index (normal range 3-7 ml/kg).

^4^ Not visible with the patient at 45º.

^5^ 1 to 2 cm above the sternal angle with the patient at 45º.

^6^ 3 to 4 cm above the sternal angle with the patient at 45º.

^7^ ≥5 cm above the sternal angle with the patient at 45º.

#### Blood pressure

On enrolment, mean (95% CI) MAP was lower in survivors than in the patients who would later die (86 mmHg (79–95) *versus* 98 mmHg (90–109)), although this was of borderline statistical significance (p = 0.051). MAP was weakly correlated with intravascular volume (r_s_ = 0.16, p = 0.002), but even in patients with significant hypovolaemia, blood pressure was usually preserved: the mean (95% CI) MAP in patients with a GEDVI <500 ml/m^2^ was 87 mmHg (84–91). MAP did not predict volume responsiveness: the mean (95% CI) MAP in volume responsive patients was 89 mmHg (82–96) *versus* 88 mmHg (83–92) in those that were not.

#### Urine output

Eight patients were anuric on admission. There was no significant difference in the mean (95% CI) GEDVI between anuric patients (516 ml/m^2^ (424–608)) and those passing urine (486 ml/m^2^ (443–529) (p = 0.58)). Six of these eight patients required renal replacement therapy (RRT), the remaining two were managed without RRT, but neither regained a urine output with fluid resuscitation. Nineteen of the 20 remaining patients were fluid resuscitated (one was in pulmonary oedema on admission) and received a median of 3.6 L in the first six hours of their hospitalization. These 19 patients had a median hourly urine output of 100 ml/hour although four subsequently developed clinical pulmonary oedema, two of whom also developed anuria in the setting of multi-organ dysfunction.

#### Other signs of hypovolaemia

Other clinical indices performed poorly in identifying hypovolaemia and predicting volume responsiveness (Tables 2, 3 and 4). Prolonged capillary refill and decreased tissue turgor occurred infrequently (7 and 4% of assessments, respectively). Patients with poor capillary return had a significantly lower intravascular volume; however patients with decreased tissue turgor actually had a significantly higher intravascular volume. Meanwhile dry axillae and dry mucous membranes were neither sensitive nor specific for hypovolaemia. Heart rate correlated weakly with volume status (r_s_ = −0.16, p = 0.002); tachycardic patients were usually hypovolaemic and heart rate fell as patients were fluid loaded early in their hospitalization. However, temperature was also elevated during this period and was also correlated with heart rate (r_s_ = 0.32, p < 0.001); while both hypovolaemia and temperature correlated with heart rate on multivariate analysis, temperature had the stronger association.

**Table 2:** Ability of clinical signs to identify hypovolaemia in adults with severe malaria

**Clinical finding**	**Hypovolaemic**^**1**^	**Euvolaemic**^**1**^	**PPV**	**NPV**	**Sensitivity**	**Specificity**
			**(95% CI)**	**(95% CI)**	**(95% CI)**	**(95% CI)**
Dry mucous membranes	235/324	42/65	85%	21%	73%	35%
			(80–89)	(14–29)	(68–78)	(24–48)
Dry axillae	138/324	27/65	84%	17%	43%	58%
			(77–89)	(12–23)	(37–48)	(46–71)
Decreased tissue turgor	12/324	7/65	63%	16%	4%	89%
			(38–84)	(12–20)	(2–6)	(79–96)
Prolonged capillary refill ^2,3^	28/307	1/53	97%	16%	9%	98%
			(82–100)	(12–20)	(6–13)	(90–100)
Tachycardia > 100 bpm^3^	127/308	16/53	89%	17%	41%	70%
			(82–93)	(12–23)	(36–47)	(56–82)
Tachycardia > 120 bpm^3^	28/308	2/53	93%	15%	9%	96%
			(78–99)	(12–20)	(6–13)	(87–100)
Low JVP^4^	101/303	18/64	85%	18%	33%	72%
			(77–91)	(14–24)	(28–39)	(59–82)

*PPV* Positive predictive value, *NPV* Negative predictive value.

^1^ Hypovolaemic: global end diastolic volume index (GEDVI) <680 ml/m^2^, euvolaemic: GEDVI: 680–800 ml/m^2^.

^2^ Greater than 2 seconds.

^3^ Excluding patients receiving inotropic support.

^4^ JVP < 3-4 cm above the sternal angle with the patient at 45º.

**Table 3:** Correlation between clinical signs of hypovolaemia and invasive haemodynamic measures

		**Number of observations**	**GEDVI**^**1**^	**CI**^**2**^	**ELWI**^**3**^
			**mean (95% CI)**	**mean (95% CI)**	**mean (95% CI)**
Mucous membranes	Normal	132	572 (548 – 595)	3.57 (3.43 – 3.71)	9.6 (8.9 – 10.3)
	Dry	297	562 (548 – 576)	3.42 (3.34 – 3.51)	9.8 (9.4 – 10.1)
Axillae	Moist	253	571 (556–585)	3.47 (3.38 -3.57)	9.4 (9.0 -9.8)
	Dry	177	556 (536–576)	3.46 (3.35 – 3.56)	10.1 (9.6 -10.7)
Tissue turgor	Normal	409	560 (548 – 573)	3.5 (3.43 - 3.57)	9.7 (9.3- 10.0)
	Decreased	21	643 (602 – 684)^ɸ^	2.83 (2.59 - 3.07) ^ɸ^	10.8 (9.3 – 12.2)
Capillary return*	Normal	371	566 (554–579)	3.52 (3.45 -3.59)	9.5 (9.2 – 9.9)
	Reduced	30	504 (473–534) ^ɸ^	2.84 (2.54 -3.14) ^ɸ^	9.9 (8.4 – 11.5)
Heart rate*	<100 bpm	269	575 (560 – 590)	3.43 (3.36 – 3.51)	9.3 (8.8 - 9.7)
	≥100 bpm	150	543 (524 – 562) ^ɸ^	3.51 (3.38 – 3.65)	10.0 (9.5 – 10.5) ^ɸ^
Heart rate*	<120 bpm	387	565 (552 – 578)	3.48 (3.41 – 3.55)	9.4 (9.1 – 9.7)
	≥120 bpm	32	528 (497 – 560)	3.3 (2.93 - 3.66)^Ʊ^	11.4 (10.2 - 12.7) ^ɸ^

^1^ Global end-diastolic volume index.

^2^ Cardiac index.

^3^ Extravascular lung water index.

* In the absence of inotropic therapy.

^ɸ^ p < 0.01 for a difference.

^Ʊ^ p < 0.05 for a difference.

**Table 4:** Ability of clinical signs to identify volume responsiveness in adults with severe malaria (Includes only those patients receiving a fluid bolus of >500 ml in the absence of inotropic support)

**Clinical finding**	**Volume responsive**	**Not volume responsive**	**PPV**	**NPV**	**Sensitivity**	**Specificity**
			**(95% CI)**	**(95% CI)**	**(95% CI)**	**(95% CI)**
Dry mucous membranes	19/23	44/58	30% (19–43)	78% (52–94)	83% (61–95)	24% (14–37)
Dry axillae	9/23	29/58	24% (11–40)	67% (51–81)	39% (20–61)	50% (37–63)
Decreased tissue turgor	2/23	2/58	50% (7–93)	73% (61–82)	9% (1–28)	97% (88–100)
Prolonged capillary refill ^1*^	4/23	12/57	25% (7–52)	70% (58–81)	17% (5–39)	79% (66–89)
Tachycardia > 100 bpm	11/23	29/58	28% (15–44)	71% (54–84)	48% (27–69)	50% (37–63)
Tachycardia > 120 bpm	0/23	3/58	0% (0–77)	71% (59–80)	0% (0–15)	95% (86–99)
Low JVP^2*^	11/23	15/57	42% (23–63)	78% (64–88)	48% (27–69)	74% (60–85)
Very low JVP^3*^	6/23	9/57	40% (16–68)	74% (61–84)	26% (10–48)	84% (72–93)

^1^ Greater than 2 seconds.

^2^ Low JVP: < 3–4 cm above the sternal angle with the patient at 45º.

^3^ Very low JVP: invisible at rest with the patient at 45º, jugular vein fills with base of neck compression.

^*^ In one patient the JVP was not visible and in one the capillary refill was not recorded.

#### Identification of pulmonary oedema

Lung water correlated with respiratory function as determined by the SaO_2_:FiO_2_ ratio (r_s_ = −0.24, p < 0.0001). A SaO_2_:FiO_2_ ratio was available on 87 of the 99 occasions when ELWI was ≥ 11 ml/kg (poor peripheral circulation precluded a reliable oximetry reading in the remaining cases). On 58 (67%) of these 87 occasions respiratory function was significantly impaired (SaO_2_:FiO_2_ ratio <450).

The JVP did not correlate with the lung water either on admission (r_s_ = −0.06, p = 0.77) or on the 367 times they were measured concurrently (r_s_ = −0.05, p = 0.31). An elevated and very low JVP had a similar PPV for pulmonary oedema (when defined as an ELWI of ≥11 ml/kg, normal range 3-7 ml/kg) (Table 1). The JVP did not correlate with respiratory function (as determined by SaO_2_:FiO_2_ ratio) either on admission (r_s_ = −0.19, p = 0.34) or on the 362 times they were measured concurrently (r_s_ = −0.08, p = 0.14). Two patients had clinical pulmonary oedema (bibasal crepitations and oxygen saturation <90%, in addition to elevated ELWI) on admission; one had a normal JVP, while the other had a low JVP. Eight other patients developed clinical pulmonary oedema during the study, at the time pulmonary oedema developed four of these patients had an elevated JVP, two had a normal JVP and in two the JVP was not discernible. A respiratory rate of less than 30 and the absence of crepitations on auscultation were moderately specific in excluding pulmonary oedema, however no clinical sign had a good PPV for the condition (Table 5).

**Table 5:** Ability of clinical signs to identify pulmonary oedema in adults with severe malaria

**Clinical finding**	Pulmonary oedema*	**No pulmonary oedema**	**PPV**	**NPV**	**Sensitivity**	**Specificity**
			**(95% CI)**	**(95% CI)**	**(95% CI)**	**(95% CI)**
Crepitations on auscultation	30/99	46/254	39% (28–51)	75% (70–80)	39% (21–40)	82% (77–86)
Tachypnoea >20 breaths/min	81/98	221/250	27% (22–32)	63% (48–77)	83% (74–90)	12% (8–16)
Tachypnoea >30 breaths/min	38/98	71/250	35% (26–45)	75% (69–80)	39% (29–49)	72% (66–77)
Elevated JVP^1#^	47/92	121/247	28% (21–35)	74% (66–80)	51% (40–62)	51% (45–57)
Low JVP^2#^	31/92	69/247	31% (22–41)	74% (68–80)	34% (24–44)	72% (66–78)

*PPV* Positive predictive value, *NPV* Negative predictive value.

* Extravascular lung water index ≥ 11 ml/kg (patients with concomitant aspiration pneumonia excluded).

^1^ JVP ≥5 cm above the sternal angle with the patient at 45º.

^2^ JVP < 3–4 cm above the sternal angle with the patient at 45º.

^#^ JVP not visible in all patients.

## Discussion

Optimization of volume status is central to the management of all critically ill populations. It is challenging in well-resourced critical care settings, let alone on general wards in the rural tropics where most adults with severe malaria will be managed. While current WHO management guidelines necessarily emphasize bedside evaluation of volume status, in this prospective study of adults with severe falciparum malaria physical examination failed to identify volume-responsive patients or those at risk of pulmonary oedema reliably. Despite few alternatives in the resource-poor setting, clinicians managing patients with severe malaria must be cautious using the physical examination to guide fluid resuscitation.

All patients in this series were hypovolaemic on enrolment and, with almost 90% having severe metabolic acidosis and over a third with significant AKI, these patients represented the population most likely to benefit from rehydration. However, it is not enough to simply “fill a patient up”; fluid resuscitation should produce a demonstrable enhancement of tissue perfusion. If clinicians are to prescribe more than maintenance fluid to an adult with severe malaria they must answer two fundamental questions: is the patient volume responsive and does the patient have incipient pulmonary oedema? These data suggest that the physical examination has a limited ability to identify either population reliably.

Clinical features of severe malaria vitiate the interpretation of many of the signs of hypovolaemia. Fever, tachypnoea and anaemia may lead to tachycardia; respiratory compensation for a metabolic acidosis may lead to dry mucous membranes; malaria-associated kidney injury may lead to oliguria; while cerebral malaria complicates assessment of cerebral perfusion and precludes the assessment of postural changes in patients’ haemodynamics.

The JVP, the clinical surrogate of the invasively measured CVP, is particularly highlighted in the WHO guidelines as a guide to intravascular volume, despite it being a notoriously difficult sign for even experienced clinicians to interpret [12,18]. More relevantly, there is a growing consensus that the CVP has a limited role in guiding fluid resuscitation in critically ill patients [19] and certainly in patients with malaria, there is little correlation between CVP and clinical endpoints, such as acidosis or pulmonary oedema [20-23]. One possibility is that the extensive myocardial sequestration seen at autopsy [24] leads to reduced ventricular compliance which would explain the disparity between the volumetric (such as the GEDVI) and pressure-based indices (such as the JVP and CVP). However, whatever the mechanism, these data suggest that relying on the JVP to identify patients who are volume responsive or at risk of pulmonary oedema is unhelpful at best and may be harmful. Other physical signs were not much more useful. Abnormal capillary refill and decreased tissue turgor occurred rarely in this series, highlighting the limited utility of these two clinical signs in adults [25,26]. Indeed paradoxically, the intravascular volume of patients with decreased tissue turgor was significantly higher than patients with normal tissue turgor. Dry axillae and dry mucous membranes were present more frequently but were non-specific findings.

Blood pressure is also a suggested resuscitation endpoint in the current WHO malaria treatment guidelines [12] and is widely used to guide fluid replacement in critically ill patients. Liberal fluid resuscitation with a target MAP of ≥65 mmHg is part of the Surviving Sepsis Guidelines bundle of care, which has led to an impressive improvement in outcomes [27,28]. Even in a resource-poor setting, prompt fluid loading reduces case fatality rates in dengue shock syndrome (DSS) to <0.2% [29]. However, unlike severe sepsis and DSS, hypotension is relatively uncommon in falciparum malaria; indeed, in large studies, patients with higher blood pressure on admission to hospital are more likely to die [30]. In this series hypotension was rare and generally occurred in the setting of severe multi-organ dysfunction at which time it was usually unresponsive to fluids or inotropes. In adults with falciparum malaria the elevation in blood pressure and the systemic vascular resistance are correlated with the amount of sequestration of parasitized erythrocytes in the microvasculature [1]. Sequestration could elevate blood pressure directly via microvascular obstruction or, more likely, indirectly by leading to endothelial dysfunction and loss of vasodilatory tone [31]. The haemolysis seen in malaria results in the release of free haemoglobin leading to nitric oxide quenching which may exacerbate this endothelial dysfunction [32].

Fluid resuscitation and anti-malarial therapy are central to the treatment of AKI, a life-threatening condition which occurs in almost half of adults with severe malaria [33]. Current WHO guidelines focus appropriately on urine output as a bedside measure of renal function, although the suggested resuscitation target of 1 ml/kg/hour is greater than is used in most other settings [12,34,35] and is problematic given the lower threshold for complications seen with fluid loading in malaria. In this study the median hourly urine output of 100 ml/hour was not very much greater than the WHO recommended figure and yet to achieve this urine output patients received very generous fluid resuscitation and over 20% developed clinical pulmonary oedema. Microvascular sequestration is prominent in patients dying with AKI and this process is relatively unaffected by even liberal fluid loading [5,8]. Thus, while it may be appropriate to administer small boluses in an attempt to correct oliguria, in cases that fail to respond early referral for renal replacement therapy should be considered given the harm that may result from continuing to unsuccessfully fluid-challenge a patient.

As pulmonary oedema in patients with severe malaria results from increased pulmonary vascular permeability, it may not be surprising that clinical signs of volume status have limited utility in its identification [36]; in this study all patients were hypovolaemic or euvolaemic (as assessed by GEDVI) at the time they developed clinical pulmonary oedema. The JVP’s ability to confirm or exclude pulmonary oedema was notably poor, although an absence of tachypnoea or crepitations on chest auscultation were more helpful in ruling the condition out.

With the physical examination’s shortcomings in defining volume status in this and other settings [37-41], what is the clinician to do? It is clearly not possible to dispense with the physical examination which remains essential in identifying disease complications and concomitant pathology [42]. Individual clinical signs of hypovolaemia may be more useful when considered collectively and cumulatively and evaluated in the context of available laboratory and radiological investigations [43,44]. However these data suggest that the physical signs of volume status need to be interpreted with caution and that fluid resuscitation is potentially dangerous even in adult patients with severe falciparum malaria who are “clinically” hypovolaemic.

The study has limitations. A relatively small number of patients were studied and the strict inclusion criteria enrolled only the most critically ill patients, the findings may not be generalizable, particularly to patients with less severe disease. Two clinicians performed the physical examination, but only one performed the examination on each occasion, potentially introducing inter-observer variability into the analysis. Except for the assessment on enrolment, the clinicians were aware of the patient’s prior progress and this may have influenced their examination, although it might be expected that this would have improved the performance of the clinical findings. The gold standard for hypovolaemia and pulmonary oedema were volumetric indices derived from transpulmonary thermodilution. Whilst this method is used widely in different critical care populations it has not been specifically validated in patients with severe malaria. Only adult patients were studied, although series of African children with severe malaria have also identified limitations of traditional bedside tests of volume status [45,46].

## Conclusions

The majority of deaths in patients with severe malaria will occur within 48 hours of their admission to hospital; to reduce mortality supportive care must be improved during this critical period. Most patients are hypovolaemic on admission and any fluid deficit will need to be replaced in time, but these data suggest that clinical examination is a poor predictor of the patient’s volume status and fluid resuscitation should proceed cautiously, even in patients who are clinically hypovolaemic.

## Competing interests

The authors declare that they have no competing interests.

## Authors’ contributions

JH was the primary investigator, lead clinician, performed the statistical analysis and wrote the first draft of the manuscript. SWKL, RP, KCM, SM, SM and SC were clinicians involved with the management of the patients. SA and MUH supervised the trial in Bangladesh. SM and SM supervised the trial in India. ND, NW and AD supervised the trial and revised the manuscript. All authors read and approved the final manuscript.
